# 1-[6-(6-Acetyl­pyridin-2-yl)pyridin-2-yl]ethanone

**DOI:** 10.1107/S160053681101556X

**Published:** 2011-05-07

**Authors:** Huseyin Zekeriya Dogan, Abdurrahman Sengul, Simon John Coles

**Affiliations:** a Department of Chemistry, Faculty of Arts and Sciences, Zonguldak Karaelmas University, TR-67100 Zonguldak, Turkey; b School of Chemistry, University of Southampton, University Road, Highfield, Southampton SO17 1BJ, England

## Abstract

In the title compound, C_14_H_12_N_2_O_2_, the asymmetric unit comprises one half-mol­ecule with an inversion center between the pyridine rings. The rings are *trans* coplanar with the acetyl groups deviating slightly from the mean planes, making a dihedral angle of 4.63 (4)°. In the crystal, mol­ecules are linked by weak inter­molecular C—H⋯O hydrogen bonds, forming a supra­molecular sheet parallel to (100).

## Related literature

The compound is of inter­est with respect to supra­molecular chemistry as a precursor for polypyridyl bridging ligands. For related structures, see: Parks *et al.* (1973 ▶); Potts *et al.* (1993 ▶); Zong *et al.* (2006 ▶); Şengül *et al.* (1998) ▶; Agac *et al.* (2010 ▶); Iyoda *et al.* (1990 ▶); Janiak *et al.* (1999 ▶); O’Donnell & Steel (2010 ▶); Kochel (2005 ▶). For applications of related structures, see: Parks *et al.* (1973 ▶); Iyoda *et al.* (1990 ▶); Şengül *et al.* (2009 ▶); Agac *et al.* (2010 ▶).
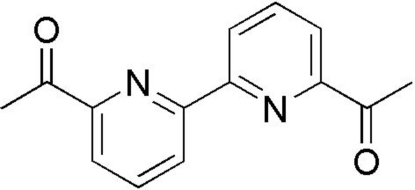


## Experimental

### 

#### Crystal data

C_14_H_12_N_2_O_2_
*M* *_r_* = 240.26
Monoclinic, 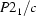

*a* = 3.9338 (2) Å
*b* = 13.8005 (8) Å
*c* = 10.8728 (6) Å
β = 94.437 (4)°
*V* = 588.50 (6) Å^3^
*Z* = 2
Mo *K*α radiation
μ = 0.09 mm^−1^
*T* = 120 K
0.50 × 0.20 × 0.20 mm

#### Data collection

Bruker–Nonius KappaCCD diffractometer with APEXII area detector
Absorption correction: multi-scan (*SADABS*; Sheldrick, 2007 ▶) *T*_min_ = 0.955, *T*_max_ = 0.982
10564 measured reflections
1336 independent reflections
1220 reflections with *I* > 2σ(*I*)
*R*_int_ = 0.034

#### Refinement

*R*[*F* ^2^ > 2σ(*F* ^2^)] = 0.042
*wR*(*F* ^2^) = 0.105
*S* = 1.10
1336 reflections
83 parameters
H-atom parameters constrained
Δρ_max_ = 0.26 e Å^−3^
Δρ_min_ = −0.19 e Å^−3^

### 

Data collection: *COLLECT* (Hooft, 1998 ▶); cell refinement: *DENZO* (Otwinowski & Minor, 1997 ▶) and *COLLECT*; data reduction: *DENZO* (Otwinowski & Minor, 1997 ▶) and *COLLECT*; program(s) used to solve structure: *SHELXS97* (Sheldrick, 2008 ▶); program(s) used to refine structure: *SHELXL97* (Sheldrick, 2008 ▶); molecular graphics: *PLATON* (Spek, 2009 ▶); software used to prepare material for publication: *publCIF* (Westrip, 2010 ▶).

## Supplementary Material

Crystal structure: contains datablocks I, global. DOI: 10.1107/S160053681101556X/bq2286sup1.cif

Structure factors: contains datablocks I. DOI: 10.1107/S160053681101556X/bq2286Isup2.hkl

Supplementary material file. DOI: 10.1107/S160053681101556X/bq2286Isup3.cml

Additional supplementary materials:  crystallographic information; 3D view; checkCIF report

## Figures and Tables

**Table 1:** Hydrogen-bond geometry (Å, °)

*D*—H⋯*A*	*D*—H	H⋯*A*	*D*⋯*A*	*D*—H⋯*A*
C3—H3⋯O1^i^	0.95	2.56	3.2992 (16)	135

Symmetry code: (i) 
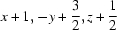
.

**Fig. 1.**
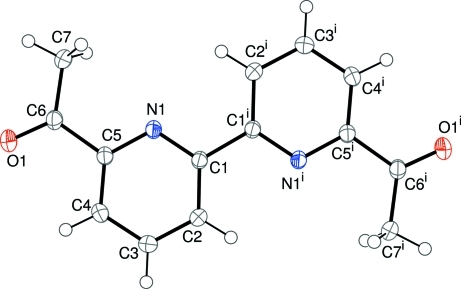
The molecular structure of (I), with the atom-numbering scheme. Displacement ellipsoids are drawn at the 50% probability level. Symmetry code: (i) -x, -y, -z

**Fig. 2.**
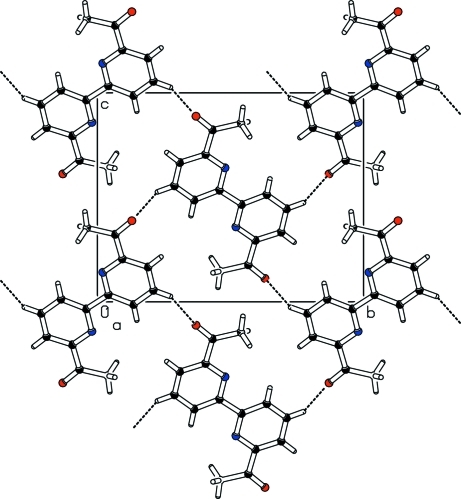
Intermolecular C=O···H contacts forming a supramolecular sheet along the a axis

## supplementary crystallographic information

### Comment

The principles of supramolecular chemistry provide guidelines for the construction of quite complex molecules or constructs from relatively simple components. In this respect, 6,6'-diacetyl-2,2'-bipyridine, acting as a diketone has been widely used as a precursor or building block for the construction of polypyridine bridging ligands [Şengül *et al.*, 2009; Agac *et al.*, 2010; Potts *et al.*, 1993; Zong *et al.*, 2006]. The well established coordination ability of 2,2'-bipyridine suggests that ligands containing multiple pyridine rings joined through their 2,6-positions would be ideal for the self-assembly of mono-, double-, or triple-stranded helicates containing one or more transition-metal cations and producing a variety of coordination geometries and architectures. This area is therefore of interest with respect to supramolecular chemistry as a precursor for polypyridyl bridging ligands (Janiak *et al.*, 1999; Potts *et al.*, 1993; Zong *et al.*, 2006) and derivatives are important materials for the preparation of oximes or other funcionalities (Iyoda *et al*., 1990; Parks *et al.*, 1973; Agac *et al.*, 2010).

As a continuation of work on the structures of such compounds (Şengül *et al.*, 1998) the title compound derived from the coupling of 6-bromo-2-acetylpyridine is reported herein. The molecule of the title compound (Fig. 1.) possesses a twofold symmetry where each of the pyridyl rings are *trans* to each other, forming an essentially planar structure. The bond lengths have normal values (Şengül *et al.*, 1998), and are comparable to those observed in similar compounds (Janiak *et al.*, 1999; O'Donnell & Steel, 2010; Kochel, 2005; Şengül *et al.* 2009).

In the crystal, molecules are linked through intermolecular C-H···O H-bonds (Table 1) to form a supramolecular network parallel to (100) (Fig. 1).

### Experimental

The title compound was synthesized by the reported method of homocoupling of aryl halides using Ni(II) complex and zinc in the presence of triphenylphosphine by Janiak *et al.* (1999). The spectroscopic and analytical data are in good agreement with the reported values in literature by Zong *et al.*, 2006; Potts *et al.*, 1993; Agac *et al.*, 2010 and Parks *et al.*, 1973. The solid was crystallized from dichloromethane to afford colourless needless suitable for X-ray diffraction. Mp.: 178.5–179.5 °C. ^1^H-NMR (dmso-*d*_6_, *δ*_p.p.m._): 8.81(d, 2H, *J*_3,4_ = 8 Hz, H3,3'), 8.23(d, 2H, *J*_5,4_ = 7 Hz, H5,5'), 8.07(dd, 2H, *J*_4,3_ = 8.2 Hz, *J*_4,5_ = 1 Hz, H4,4'), 2.79(s, 6H, 2xCH_3_). *Calc*. for C_14_H_12_N_2_O_2_: C, 69,99; H, 5,03; N, 11,66 Found: C,62,54; H, 4,54; N, 11,68%. IR (ATR, *ν* cm^-1^): 3056 (CH_ar_), 2990 (CH_al_), 1590 (C=O), 1487 and 1437 (C=N and C=C), 1311, 1182, 1120, 1094, 1071, 995, 861, 748, 720. UV-*Vis* (MeCN, *λ*_max_/nm): 286, 258, 219.

### Refinement

Hydrogen atoms were fixed in idealized positions [0.98 Å (CH_3_) & 0.95 Å (CH)] and refined using the riding model with *U*_ĩso_ (H) set to 1.5 and 1.2*U*_eq_(carrier) respectively.

### Crystal data

**Table:**

C_14_H_12_N_2_O_2_	*F*(000) = 252
*M**_r_* = 240.26	*D*_x_ = 1.356 Mg m^−^^3^
Monoclinic, *P*2_1_/*c*	Melting point: 452 K
Hall symbol: -P 2ybc	Mo *K*α radiation, λ = 0.71073 Å
*a* = 3.9338 (2) Å	Cell parameters from 10564 reflections
*b* = 13.8005 (8) Å	θ = 2.9–27.5°
*c* = 10.8728 (6) Å	µ = 0.09 mm^−^^1^
β = 94.437 (4)°	*T* = 120 K
*V* = 588.50 (6) Å^3^	Rod, colourless
*Z* = 2	0.50 × 0.20 × 0.20 mm

### Data collection

**Table:**

Bruker–Nonius Kappa CCD diffractometer with APEXII area detector	1336 independent reflections
Radiation source: Bruker-Nonius FR591 rotating anode	1220 reflections with *I* > 2σ(*I*)
10cm confocal mirrors	*R*_int_ = 0.034
φ and ω scans	θ_max_ = 27.5°, θ_min_ = 3.8°
Absorption correction: multi-scan (*SADABS*; Sheldrick, 2007)	*h* = −5→4
*T*_min_ = 0.955, *T*_max_ = 0.982	*k* = −17→17
10564 measured reflections	*l* = −14→14

### Refinement

**Table:**

Refinement on *F*^2^	Primary atom site location: structure-invariant direct methods
Least-squares matrix: full	Secondary atom site location: difference Fourier map
*R*[*F*^2^ > 2σ(*F*^2^)] = 0.042	Hydrogen site location: inferred from neighbouring sites
*wR*(*F*^2^) = 0.105	H-atom parameters constrained
*S* = 1.10	*w* = 1/[σ^2^(*F*_o_^2^) + (0.0431*P*)^2^ + 0.237*P*] where *P* = (*F*_o_^2^ + 2*F*_c_^2^)/3
1336 reflections	(Δ/σ)_max_ = 0.001
83 parameters	Δρ_max_ = 0.26 e Å^−^^3^
0 restraints	Δρ_min_ = −0.19 e Å^−^^3^

### Fractional atomic coordinates and isotropic or equivalent isotropic displacement parameters (Å^2^)

**Table:**

	*x*	*y*	*z*	*U*_iso_*/*U*_eq_	
C1	0.4161 (3)	0.54168 (8)	0.46613 (10)	0.0185 (3)	
C2	0.4431 (3)	0.63598 (9)	0.51269 (11)	0.0234 (3)	
H2	0.5670	0.6485	0.5895	0.028*	
C3	0.2863 (3)	0.71096 (9)	0.44508 (12)	0.0277 (3)	
H3	0.3018	0.7757	0.4748	0.033*	
C4	0.1063 (3)	0.69010 (9)	0.33338 (12)	0.0248 (3)	
H4	−0.0011	0.7402	0.2846	0.030*	
C5	0.0871 (3)	0.59397 (8)	0.29474 (10)	0.0197 (3)	
C6	−0.1175 (3)	0.56675 (9)	0.17703 (11)	0.0216 (3)	
C7	−0.1483 (3)	0.46102 (9)	0.14615 (11)	0.0247 (3)	
H7A	−0.2969	0.4528	0.0703	0.037*	
H7B	−0.2460	0.4266	0.2139	0.037*	
H7C	0.0780	0.4346	0.1341	0.037*	
N1	0.2396 (2)	0.52066 (7)	0.35884 (9)	0.0190 (2)	
O1	−0.2556 (3)	0.62953 (7)	0.11205 (8)	0.0315 (3)	

### Atomic displacement parameters (Å^2^)

**Table:**

	*U*^11^	*U*^22^	*U*^33^	*U*^12^	*U*^13^	*U*^23^
C1	0.0184 (5)	0.0192 (6)	0.0174 (5)	−0.0012 (4)	−0.0011 (4)	0.0013 (4)
C2	0.0270 (6)	0.0206 (6)	0.0215 (6)	−0.0012 (5)	−0.0047 (5)	−0.0012 (4)
C3	0.0333 (7)	0.0185 (6)	0.0297 (7)	0.0004 (5)	−0.0073 (5)	−0.0017 (5)
C4	0.0275 (6)	0.0199 (6)	0.0259 (6)	0.0018 (5)	−0.0053 (5)	0.0032 (5)
C5	0.0199 (6)	0.0200 (6)	0.0189 (5)	0.0005 (4)	−0.0012 (4)	0.0018 (4)
C6	0.0210 (6)	0.0234 (6)	0.0199 (6)	0.0019 (4)	−0.0019 (4)	0.0016 (4)
C7	0.0258 (6)	0.0245 (6)	0.0225 (6)	0.0006 (5)	−0.0063 (5)	−0.0017 (5)
N1	0.0191 (5)	0.0197 (5)	0.0178 (5)	0.0000 (4)	−0.0014 (4)	0.0016 (4)
O1	0.0384 (6)	0.0282 (5)	0.0259 (5)	0.0068 (4)	−0.0104 (4)	0.0033 (4)

### Geometric parameters (Å, °)

**Table:**

C1—N1	1.3423 (15)	C4—H4	0.9500
C1—C2	1.3975 (16)	C5—N1	1.3433 (14)
C1—C1^i^	1.492 (2)	C5—C6	1.5058 (16)
C2—C3	1.3861 (17)	C6—O1	1.2189 (15)
C2—H2	0.9500	C6—C7	1.5000 (17)
C3—C4	1.3878 (17)	C7—H7A	0.9800
C3—H3	0.9500	C7—H7B	0.9800
C4—C5	1.3919 (17)	C7—H7C	0.9800
			
N1—C1—C2	122.39 (11)	N1—C5—C6	116.16 (10)
N1—C1—C1^i^	116.22 (12)	C4—C5—C6	120.48 (10)
C2—C1—C1^i^	121.39 (13)	O1—C6—C7	122.48 (11)
C3—C2—C1	119.01 (11)	O1—C6—C5	120.02 (11)
C3—C2—H2	120.5	C7—C6—C5	117.49 (10)
C1—C2—H2	120.5	C6—C7—H7A	109.5
C2—C3—C4	119.03 (11)	C6—C7—H7B	109.5
C2—C3—H3	120.5	H7A—C7—H7B	109.5
C4—C3—H3	120.5	C6—C7—H7C	109.5
C3—C4—C5	118.28 (11)	H7A—C7—H7C	109.5
C3—C4—H4	120.9	H7B—C7—H7C	109.5
C5—C4—H4	120.9	C1—N1—C5	117.92 (10)
N1—C5—C4	123.35 (11)		

Symmetry codes: (i) −*x*+1, −*y*+1, −*z*+1.

### Hydrogen-bond geometry (Å, °)

**Table:**

*D*—H···*A*	*D*—H	H···*A*	*D*···*A*	*D*—H···*A*
C3—H3···O1^ii^	0.95	2.56	3.2992 (16)	135.

Symmetry codes: (ii) *x*+1, −*y*+3/2, *z*+1/2.
